# Comparative genomics reveals a core gene toolbox for lifestyle transitions in Hypocreales fungi

**DOI:** 10.1111/1462-2920.15554

**Published:** 2021-05-11

**Authors:** Baojun Wu, Murray P. Cox

**Affiliations:** ^1^ Statistics and Bioinformatics Group, School of Fundamental Sciences Massey University Palmerston North 4410 New Zealand; ^2^ Bio‐Protection Research Centre Massey University Palmerston North 4410 New Zealand

## Abstract

Fungi have evolved diverse lifestyles and adopted pivotal new roles in both natural ecosystems and human environments. However, the molecular mechanisms underlying their adaptation to new lifestyles are obscure. Here, we hypothesize that genes shared across all species with the same lifestyle, but absent in genera with alternative lifestyles, are crucial to that lifestyle. By analysing dozens of species within four genera in a fungal order, with each genus following a different lifestyle, we find that genus‐specific genes are typically few in number. Notably, not all genus‐specific genes appear to derive from de novo birth, with most instead reflecting recurrent loss across the fungi. Importantly, however, a subset of these genus‐specific genes are shared by fungi with the same lifestyle in quite different evolutionary orders, thus supporting the view that some genus‐specific genes are necessary for specific lifestyles. These lifestyle‐specific genes are enriched for key functional classes and often exhibit specialized expression patterns. Genus‐specific selection also contributes to lifestyle transitions, and is especially associated with intensity of pathogenesis. Our study, therefore, suggests that fungal adaptation to new lifestyles often requires just a small number of core genes, with gene turnover and positive selection playing complementary roles.

## Introduction

Fungi occupy a broad range of ecological niches, and their activities have profound impacts on the natural environment, human health and the economy. Several lifestyles have been documented in fungi. Saprotrophic fungi decompose organic material (Floudas *et al*., 2012), pathogens take advantage of animals, plants and insects and fungal symbionts form commensal or mutualistic relationships, often with plants (Druzhinina *et al*., 2011; Fisher *et al*., 2012; Gruber and Seidl‐Seiboth, 2012; Deshmukh *et al*., 2014). In addition, some fungi have also been reported as being parasites of other fungi, namely, mycoparasitism (Barnett, 1963; Lorito *et al*., 1998; Shang *et al*., 2015; Karlsson *et al*., 2017). Lifestyle transitions across the fungi are widespread and recurrent (Naranjo‐Ortiz and Gabaldon, 2019b). However, it remains unclear how fungi evolve their diverse lifestyles during these transitions. Most insights so far have come from studies of individual genes (Brotman *et al*., 2008; Lopez‐Berges *et al*., 2010; Koch *et al*., 2013; Schardl *et al*., 2013; Gomes *et al*., 2015; Berry *et al*., 2019; Dilks *et al*., 2019; Estrada‐Rivera *et al*., 2019; Vikuk *et al*., 2019; Wu and Cox, 2019) or analyses where a lifestyle is represented by a single species (Mendoza‐Mendoza *et al*., 2003; Le Crom *et al*., 2009; Reithner *et al*., 2011; Hernandez‐Onate *et al*., 2012; Wiemann *et al*., 2013; Zhang *et al*., 2016; Dinkins *et al*., 2017; Schirrmann *et al*., 2018; Chen *et al*., 2019; Wu and Cox, 2019; Zhang *et al*., 2020). However, genes identified from a single species can be conflated with species‐specific adaptation, such as to a specific host, rather than the broader question of the origin of a particular lifestyle. In other words, these earlier analyses ignore the considerable variation observed among species with the same lifestyle, and thus are unlikely to identify the core gene toolbox related to that specific lifestyle.

The fungal order Hypocreales provides a natural resource to study transitions to new lifestyles. This order includes crop pathogens in the genus *Fusarium* (Ma *et al*., 2013), pasture grass symbionts in *Epichloë* (Saikkonen *et al*., 2016), ubiquitous insect pathogens in *Metarhizium* (Branine *et al*., 2019) and mycoparasites in *Trichoderma* (Mukherjee *et al*., 2013). Four different lifestyles in the same fungal order, together with a rich array of species in each lifestyle, make the Hypocreales an excellent model to investigate the broader question of how fungi evolve different lifestyles over a relatively short evolutionary time.

We hypothesize that genes shared across all species with the same lifestyle, but absent in genera with alternative lifestyles, are likely to be crucial for that lifestyle. To test this hypothesis, we investigate genus‐specific genes and their role in lifestyle transitions among four genera in the order Hypocreales. Although genus‐specific genes are few in number, these genes often appear in other fungi from quite different evolutionary orders with the same lifestyle, and thus are likely to be functionally associated with specific lifestyles.

## Results

### Genus‐specific genes are few in number

**Fig 1:**
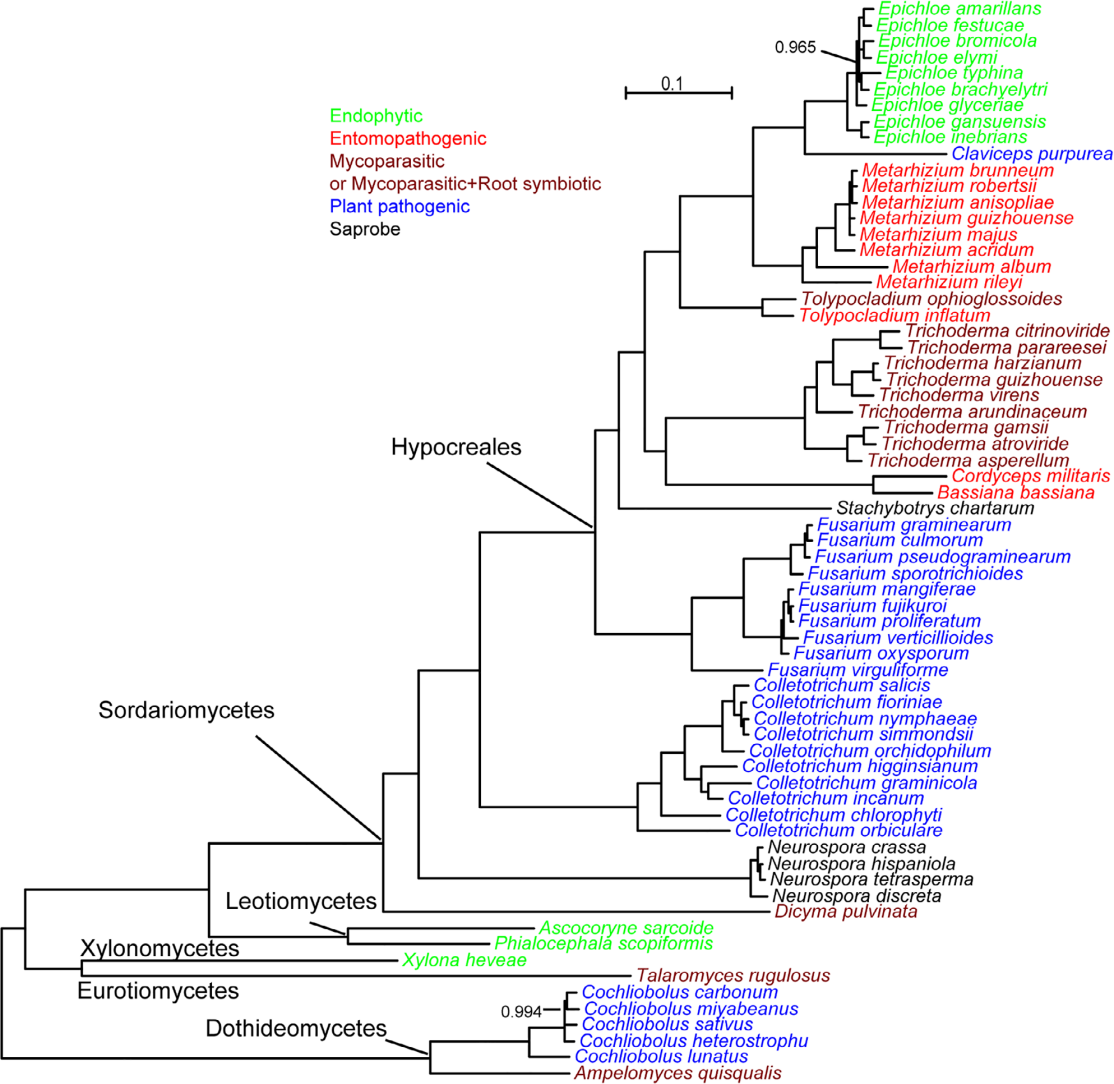
Maximum likelihood phylogeny of taxa analysed in this study. A RAxML‐NG tree of 1018 proteins identified by Orthofinder. Taxa are coloured by lifestyle. *Trichoderma* has two main lifestyles, as does *Talaromyces rugulosus*. Phylogenetic trees generated by FastTree 2 and RAxML‐NG are topologically identical, so only the tree constructed with RAxML‐NG is shown. All bootstrap values smaller than 100 are shown. Species from the Dothideomycetes family were used as outgroups. [Color figure can be viewed at wileyonlinelibrary.com]

**Fig 2:**
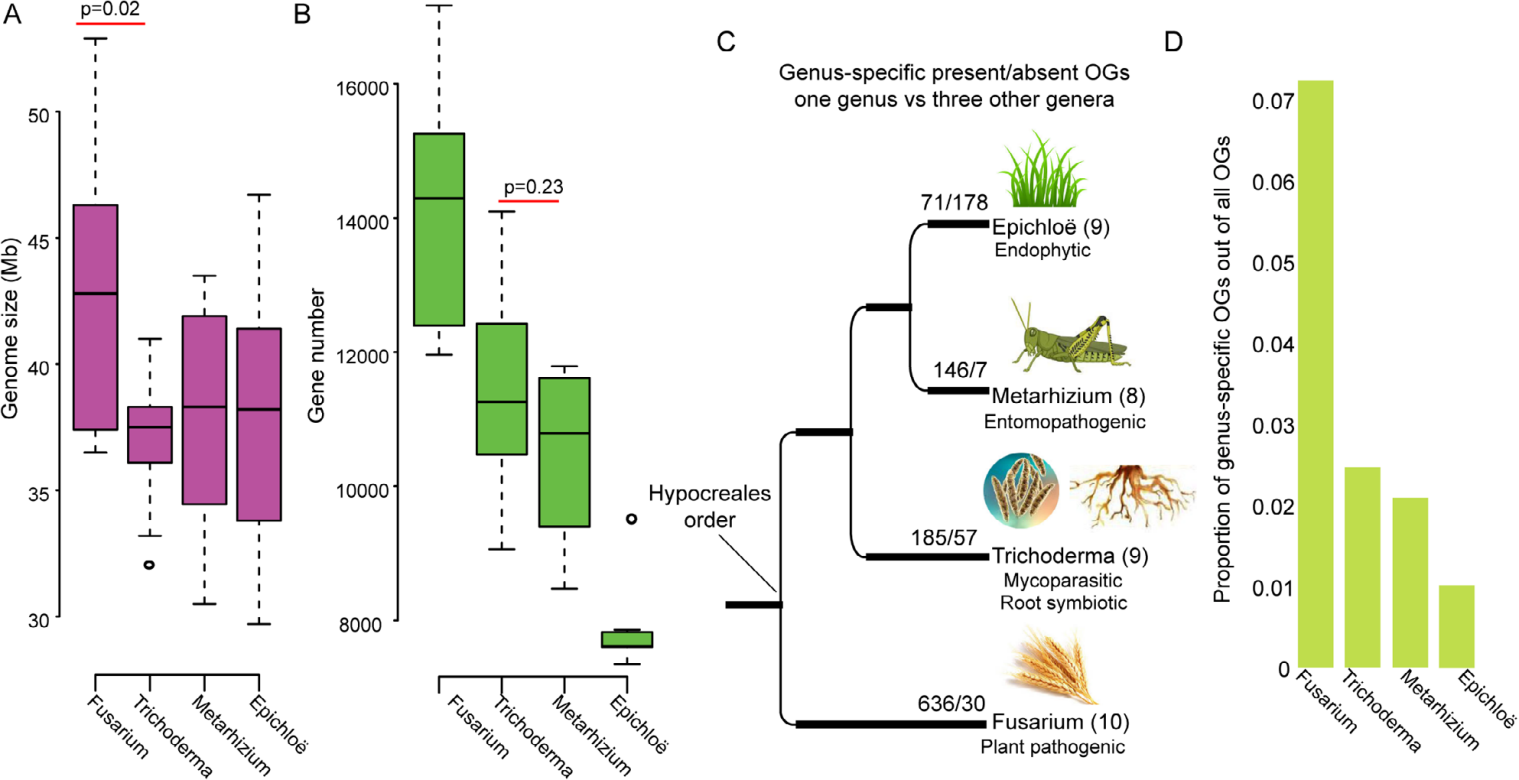
Genome size, gene number and gene turnover in each genus. A. Comparison of genome assembly size among four genera. B. Comparison of gene number among four genera. Statistical significance was determined with the Mann–Whitney *U* test. The genera are ordered relative to their phylogenetic relationships. C. Genus‐specific present and absent orthogroups (OGs). Representative hosts are illustrated above each genus. Numbers in parentheses indicate the number of species studied in each genus. D. Proportion of genus‐specific present OGs in individual species. Only the highest proportion in each genus is shown. [Color figure can be viewed at wileyonlinelibrary.com]

Although the concept of fungal lifestyles is in some ways a human one, this framing has been widely used in fungal studies (O'Connell *et al*., 2012; Knapp *et al*., 2018; Naranjo‐Ortiz and Gabaldon, 2019a; Miyauchi *et al*., 2020). Twelve lifestyles have been proposed for fungi (Naranjo‐Ortiz and Gabaldon, 2019a). In this study, we focused on four major lifestyles: endophytic (plant‐associated), entomopathogenic (animal‐associated), mycoparasitic (fungi‐associated) and plant pathogenic (Fig. 1). We studied four genera, each with a different lifestyle, to identify the molecular underpinnings of lifestyle transitions. The four genera include the crop pathogens *Fusarium* (Ma *et al*., 2013), pasture grass symbionts *Epichloë* (Saikkonen *et al*., 2016), insect pathogens *Metarhizium* (Branine *et al*., 2019) and mycoparasites *Trichoderma* (Mukherjee *et al*., 2013). Each genus includes 8–10 species‐level genomes with fewer than 5% missing BUSCO genes (Fig. 1 and Table S1). Although genome assembly size does not differ significantly among the four genera (Fig. 2A), gene number has experienced significant reductions in some lifestyles (Fig. 2B).

Orthofinder assigned 731,739 proteins from 67 species to 25,005 orthogroups, which is defined as the set of genes that descend from a single gene in the last common ancestor of all the species being considered (Emms and Kelly, 2015). We examine expansion and contraction of gene families (orthogroup copy number) at four branches leading to four genera. Of note, 37 (minimum number among four genera) to 584 (maximum number among four genera) orthogroups show signs of expansion, while 19–148 orthogroups contracted at the same branches (Fig. S1). However, only 0–20 orthogroups show statistically significant expansion or contraction using CAFE 5 (Mendes *et al*., 2020) (Fig. S1 and Table S2). Except for *Epichloë*, expansion of gene families is more common than contraction (Fig. S1). We also identify 2–37 duplicates that are present in only one genus (Fig. S1 and Table S3). These genes are enriched for proteins involved in transmembrane transport, oxidation–reduction processes and integral components of membranes (Tables S2 and S3).

To further track down the genes specific to each genus, which in turn are expected to be important for lifestyle transitions, we identified genus‐specific ‘present’ and ‘absent’ orthogroups. Our study assumes that absence of an orthogroup within some species in a genus is suggestive of a non‐crucial role for that gene in the genus' lifestyle. Classification as a genus‐specific gene requires that the orthogroup must be present in all analysed species in the genus (or absent only once to accommodate some level of genome assembly error) and absent in every species in the other genera and vice versa for genus‐specific absence genes. Consequently, we identified 71–636 genus‐specific present and 7–178 genus‐specific absent genes respectively (Fig. 2C and Table S4 and S5). We also checked the number of genes per orthogroup and found that most of the genus‐specific orthogroups only have a single‐copy gene. Genus‐specific genes are very few in number, representing only 1%–7% of all orthogroups (Fig. 2D). Given great variation in gene number within and between genera (Fig. 2B), this pattern indicates that most gene turnover in the four genera is species‐specific rather than genus‐specific, thus showing the importance of our study design, which analyses multiple species for each genus and lifestyle (Fig. S2).

### Not all genus‐specific present genes are new genes

Where did genus‐specific genes come from? Three possibilities exist: they are new genes; independent loss events have occurred across fungi; and horizontal gene transfer (HGT). To quantify these alternatives, we examined the possible origins of genus‐specific genes by BLAST search against 190 *Sordariomycetes* genomes representing nine fungal orders and the NCBI nr database, which represents diversity across the tree‐of‐life. We assigned genus‐specific genes to these three alternative mechanisms on the basis of the BLAST results: (i) independent loss events, if genus‐specific present genes have homologues in other orders in the *Sordariomycetes* family; (ii) HGT, if genus‐specific present genes lack homologues in the *Sordariomycetes* family but have homologues in other organisms and (iii) new genes if genus‐specific present genes do not belong to the two previous categories.

**Fig 3:**
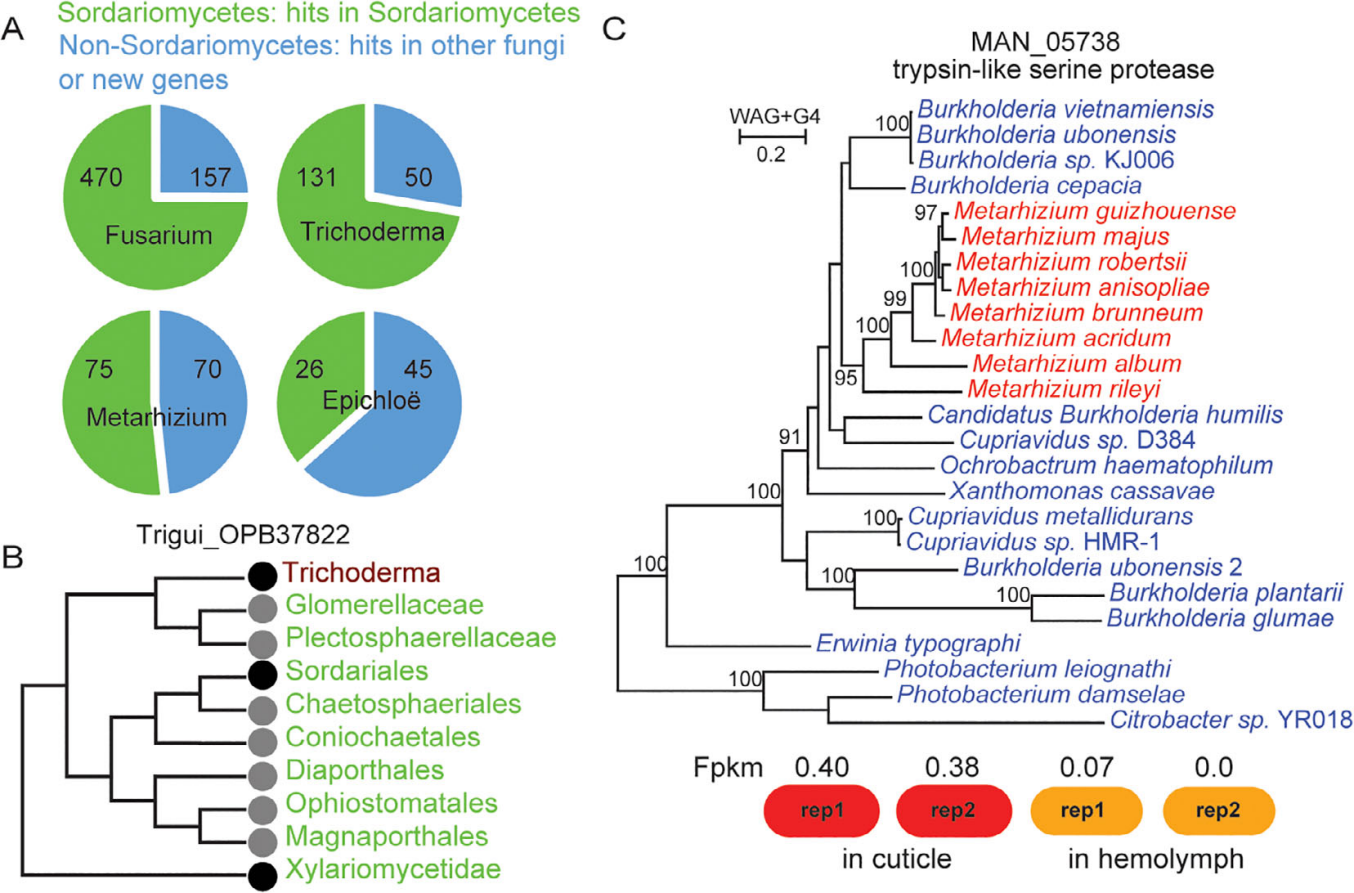
Origins of genus‐specific present genes. A. Distribution of genus‐specific present genes. ‘Sordariomycetes’ refers to homologues found in other members of the Sordariomycetes family, while ‘non‐Sordariomycetes’ refers to genus‐specific genes not found in other members of the Sordariomycetes family and either acquired by horizontal gene transfer from bacteria/fungi or which arose as new genes. The BLAST search was performed using *Fusarium graminearum*, *Trichoderma virens*, *Metarhizium anisopliae* and *Epichloë festucae*. B. A case of independent gene loss of a genus specific orthogroup in the *Trichoderma* genus among different orders in the *Sordariomycetes* family. Black circle: presence; Grey circle: absence. C. A case of horizontal gene transfer shaping a genus‐specific present gene MAN_05738 of *Metarhizium* (red) from a soil bacterium in the genus *Burkholderia (*blue – all bacteria). The expression level of the gene during insect infection is shown as FPKM (Fragments Per Kilobase of transcript per Million mapped reads). The ‘in cuticle’ and ‘in hemolymph’ conditions are two stages of insect infection. [Color figure can be viewed at wileyonlinelibrary.com]

Over 37% of genus‐specific present genes in Hypocreales have homologues in other orders from the *Sordariomycetes* family (Fig. 3A), which is suggestive of recurrent gene gain and loss within *Sordariomycetes* (one exemplar case is shown in Fig. 3B). The genus‐specific genes without *Sordariomycetes* hits account for 25% to 63% of such genes (Fig. 3A), which are possibly derived by HGT between distant organisms or are new genes. Through BLAST searches against the NCBI nr database using genus‐specific present genes without *Sordariomycetes* hits, we find three potential inter‐kingdom HGT cases, including MAN_05738 (trypsin‐like serine protease), MAN_07610 (GH43) and FGSG_01491, which only account for fewer than 1% of genes in each genus. In other words, the number of genus‐specific genes classified as new genes far outnumber those derived by HGT. We phylogenetically constructed an HGT case in *Metarhizium*, which is the best example based on BLAST results (100% coverage and 72% identity). The phylogeny indicates that the genus‐specific present gene in *Metarhizium* was likely transferred from a soil bacterium in the genus *Burkholderia* (Fig. 3C). A shared habitat likely promoted HGT because mycelium of *Metarhizium* colonizes the soil through insect larvae feeding on root tissue (Behie *et al*., 2012; Branine *et al*., 2019). Although the transferred gene is expressed at a very low level, recent experimental work has suggested a functional role for this gene (trypsin‐like serine protease) in degrading insect cuticles (Zhang *et al*., 2019).

### Genus‐specific genes are shared by other fungal genera and species with the same lifestyle

**Fig 4:**
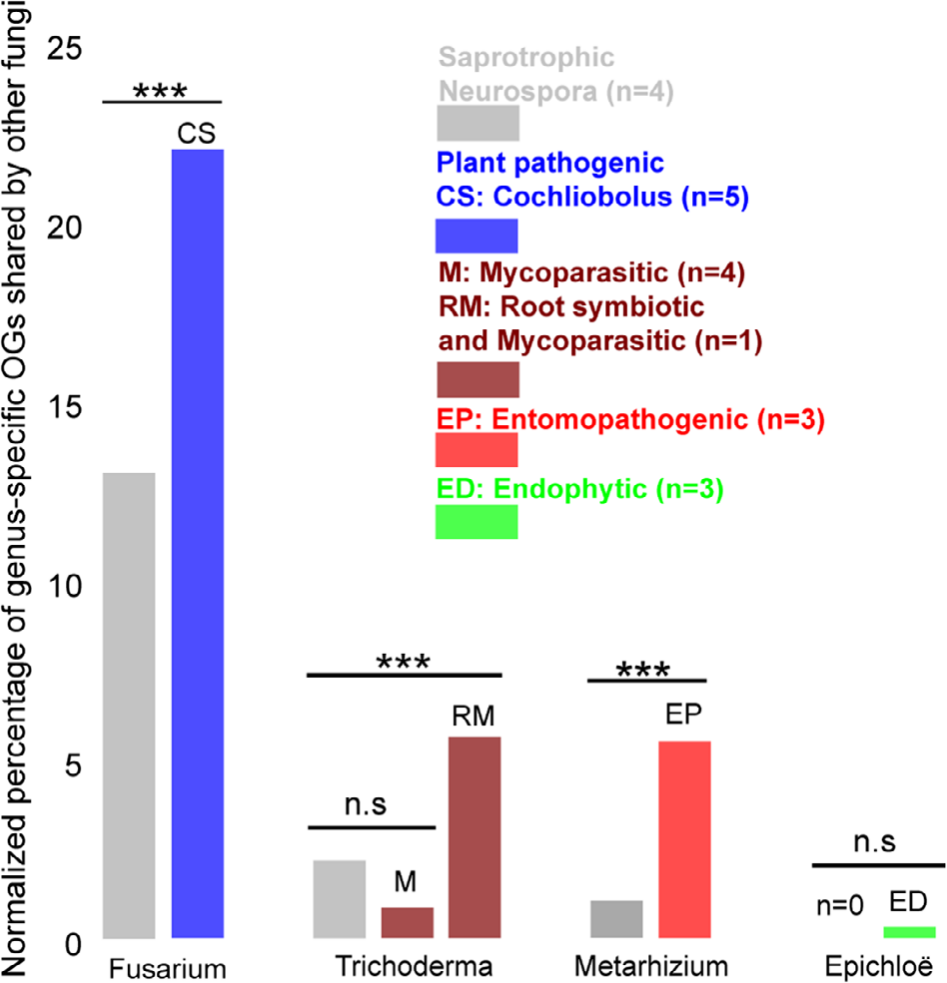
Proportion of genus‐specific orthogroups (OGs) shared by fungi in other orders with the same and different lifestyles. OGs in these other genera/species were required to be present in all species in the genus (or absent only once), or present in all fungi with the same lifestyle (or absent only once). The proportion of genus‐specific OGs against genus OGs of saprotrophic *Neurospora* (representing a different lifestyle) is a control. The percentage was normalized by shared total orthogroups between fungi. The lists in the panel on the right indicate lifestyles and species used for the comparison, while numbers in parentheses represent the number of species used for each comparison. Statistical significance was determined with the two‐sided Fisher test (*P ≤ 0.05, **P ≤ 0.001 and ***P ≤ 0.0001). [Color figure can be viewed at wileyonlinelibrary.com]

The origin of genus‐specific present genes suggests a recurrent gain and loss process. To test whether genus‐specific present genes are recurrently shared by different fungi with similar lifestyles, we compared genus‐specific orthogroups in a wide range of other fungal genera and species with similar lifestyles (Fig. 1). The proportion of genus‐specific orthogroups against genus orthogroups of saprotrophic *Neurospora* acts as a control. We find that fungi with similar lifestyles share more orthogroups than those with different lifestyles for plant pathogenic, entomopathogenic and endophytic lifestyles (Fig. 4). *Trichoderma* is an unusual exception because it has two main lifestyles, root symbiosis and mycoparasitism, and *Trichoderma* species also tend towards being generalists. When only considering the mycoparasitic lifestyle (*Tolypocladium ophioglossoides, Dicyma pulvinate, Talaromyces rugulosus* and *Ampelomyces quisqualis*), fewer genus‐specific orthogroups are shared among fungi with a mycoparasitic lifestyle than those with different lifestyles (Fig. 4). However, when taking into account both lifestyles (*T. rugulosus*), fungi with root symbiotic and mycoparasitic lifestyles share more genus‐specific orthogroups than those with different lifestyles. This pattern suggests that *Trichoderma*‐specific genes may be more characteristic of root symbiosis than mycoparasitism.

### Roles of genus‐specific present genes are correlated with fungal lifestyles

**Fig 5:**
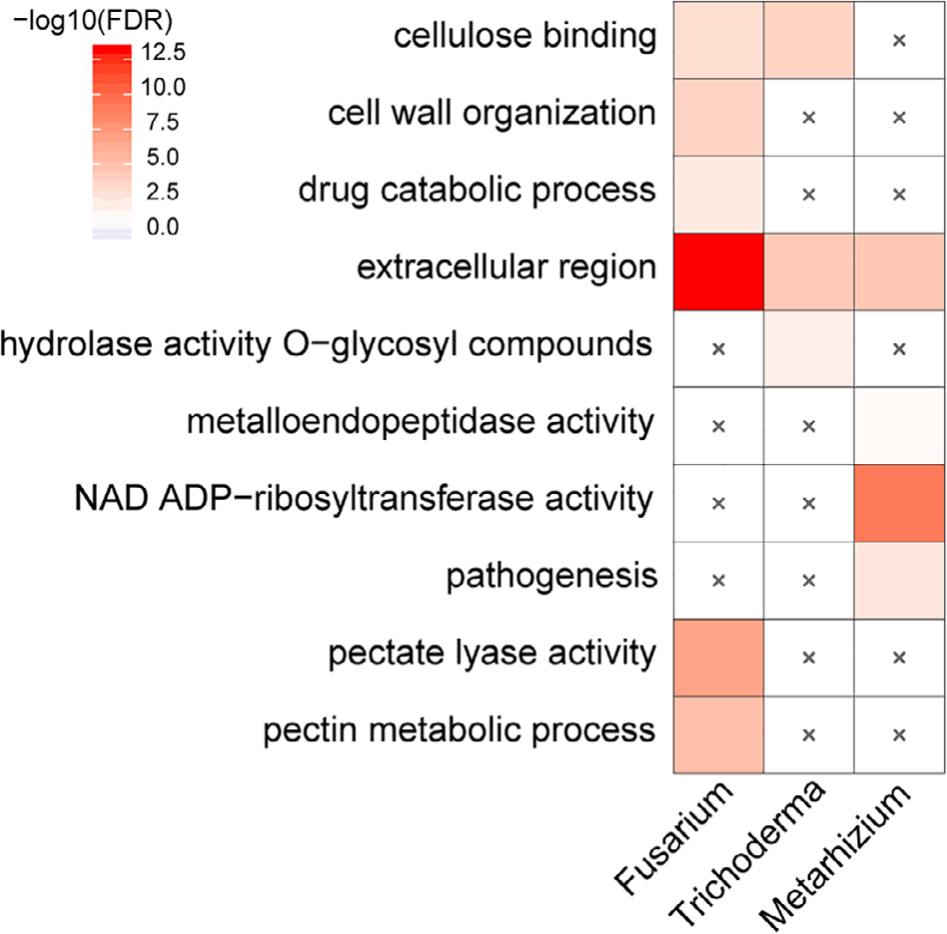
Significantly enriched GO terms of genus‐specific present genes. Significant enrichments at a corrected p value threshold of 0.05 are plotted. The enriched GO terms were performed in *Fusarium graminearum*, *Trichoderma virens*, *Metarhizium anisopliae* and *Epichloë festucae*, which have corresponding expression data for later analyses (see Fig. 6). [Color figure can be viewed at wileyonlinelibrary.com]

To screen for functional signatures among genus‐specific genes, we tested for overrepresentation of functional categories using a GO enrichment analysis. We can annotate 36–45% of the genus‐specific genes with GO terms for *Fusarium*, *Metarhizium* and *Trichoderma*, while only 14% of the genus‐specific present genes can be annotated with GO terms in *Epichloë*. Significant GO enrichments in the three genera except *Epichloë* were found (Table S6). The lack of enriched GO terms in *Epichloë* is likely due to its much smaller number of annotations. To confirm this finding, we also ran Wei2GO on the *Epichlöe‐*specific genes. Although the ratio of genus‐specific genes with annotations increased to 25%, no significantly enriched GO terms were found. This pattern suggests that the genus‐specific genes within this clade are very likely *de novo* genes, as suggested by Fig. 3A. This analysis also emphasizes several enriched functions (Fig. 5):

- In plant pathogens, genus‐specific present genes are significantly enriched for plant cell wall degradation, including ‘pectin metabolic processes’, ‘cellulose binding’ and ‘pectate lyase activity’ (Fig. 5). Plant pathogens often differentiate a dome‐shaped appressorium cell to penetrate their hosts and the appressorium has a specialized cell wall (Doehlemann *et al*., 2017). Consistent with this, the GO term ‘cell wall organization’ is also significantly enriched (Fig. 5). Interestingly, the GO term ‘drug catabolic process’ is also enriched in plant pathogens, and may be correlated with chemical resistance.
- In mycoparasitic/root symbiotic fungi, the GO terms ‘cellulose binding’ and ‘hydrolase activity acting on glycosyl bonds’ are enriched (Fig. 5). Cellulose can facilitate fungi to degrade plant cell walls and colonize living plant roots (Balestrini and Bonfante, 2014), while hydrolase activity acting on glycosyl bond can help mycoparasitic fungi to degrade the cell walls of other fungi, which are made of glycosyl units (Kang *et al*., 2018; Ruiz‐Herrera and Ortiz‐Castellanos, 2019).
- In entomopathogenic fungi, the enriched GO terms for genus‐specific present genes reflect their adaptation to insects (Fig. 5), such as ‘pathogenesis’ and ‘metalloendopeptidase activity’. For instance, metalloproteinases in *Metarhizium* have been suggested to degrade host‐derived defence molecules (Mukherjee and Vilcinskas, 2018).

Although lifestyles are very different in the three genera, the cellular component ‘extracellular region’ is enriched in all of them (Fig. 5). This suggests that genus‐specific present genes tend to be enriched for extracellular functions and are involved in the fungus interacting with its environment through secreted proteins and effectors. Although not significant, the subcellular location of genus‐specific genes in *Epichloë* tends to occur at membranes, possibly related to its endophytic lifestyle.

### Genus‐specific present genes are more likely to be up‐regulated in response to hosts

**Fig 6:**
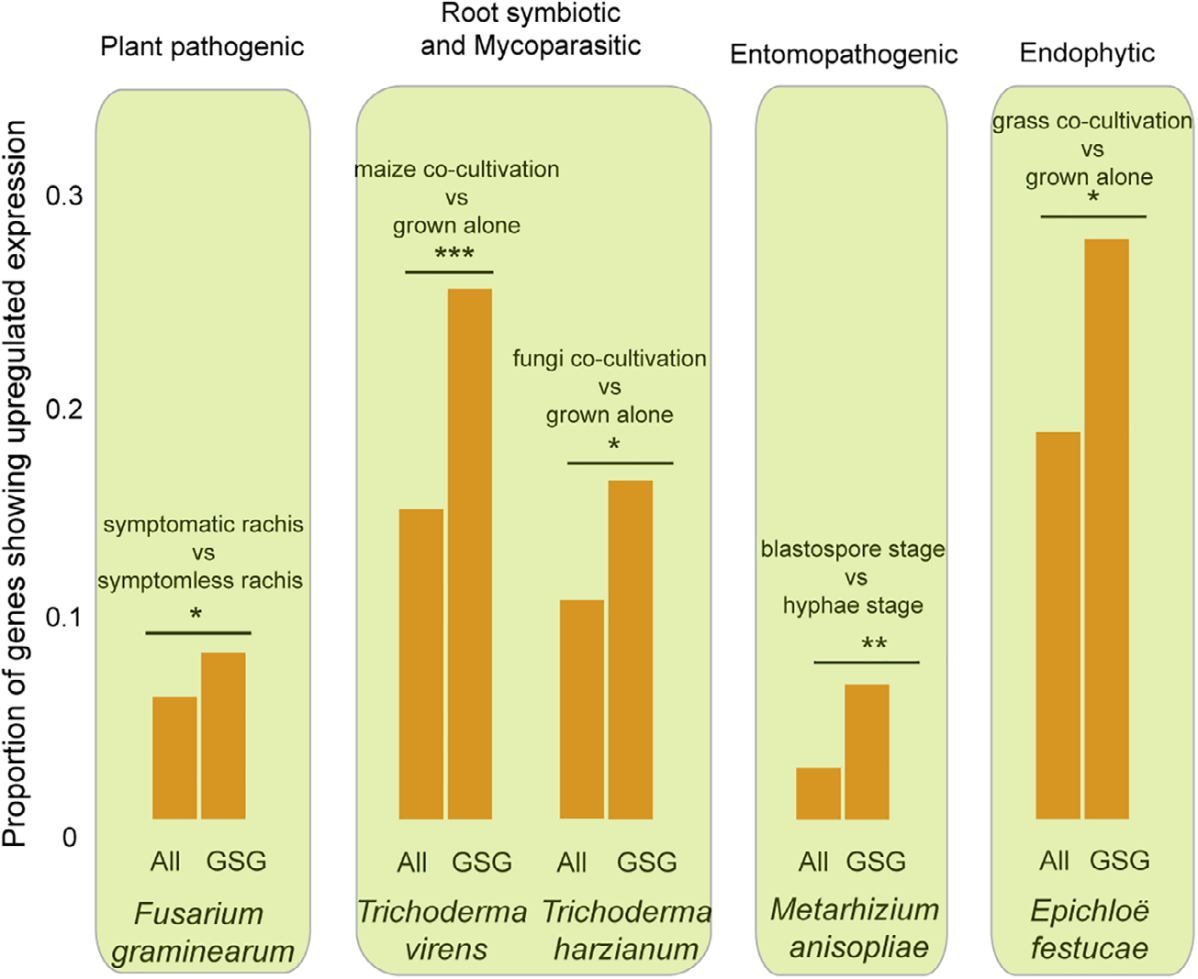
Up‐regulated expression analysis of genus‐specific present genes in (A) *Fusarium*, (B) *Trichoderma*, (C) *Metarhizium* and (D) *Epichloë*. In each panel, the histogram represents the proportion of genus‐specific present genes (GSG) and all genes (All) in the same genome that show up‐regulated expression in selected species. Statistical significance was determined with the two‐sided Fisher test (*P ≤ 0.05, **P ≤ 0.001 and ***P ≤ 0.0001). Conditions in which the gene is upregulated are labelled above the histograms. [Color figure can be viewed at wileyonlinelibrary.com]

Gene expression data allows another test of whether genus‐specific present genes are potentially functional relative to lifestyles. In particular, we test whether genes that are significantly differentially expressed during interactions with hosts are more likely to be genus‐specific present genes. As *Fusarium* is a plant pathogen, we examined gene expression patterns in a symptomatic rachis relative to a symptomless rachis. We find that up‐regulated genes are significantly more likely to be genus‐specific present genes (Fig. 6). We also examined expression patterns in plant or fungi host co‐cultivation relative to the fungus grown alone for *Trichoderma virens* (root symbiotic), *Trichoderma harzianum* (mycoparasitic) and *Epichloë festucae* (endophytic lifestyles). Consistent with observations in *Fusarium*, up‐regulated genes are significantly more likely to be genus‐specific genes (Fig. 6). For the entomopathogenic lifestyle, we examined expression patterns at the blastospore stage relative to the hyphae stage of *Metarhizium anisopliae*. Blastospores are able to penetrate insect cuticles and proliferate in the hemocoel, and blastospores have been found to be more virulent against susceptible hosts compared with aerial conidia (Alkhaibari *et al*., 2016). We find that genus‐specific genes are more often upregulated in the blastospore stage relative to hyphae (Fig. 6). This correlation between gene expression and genus‐specific present genes, observed across very different biological systems and environmental settings, strengthens our hypothesis that genus‐specific genes are functionally linked to lifestyle transitions.

### Genus‐specific absent genes reflect changes of lifestyle

**Fig 7:**
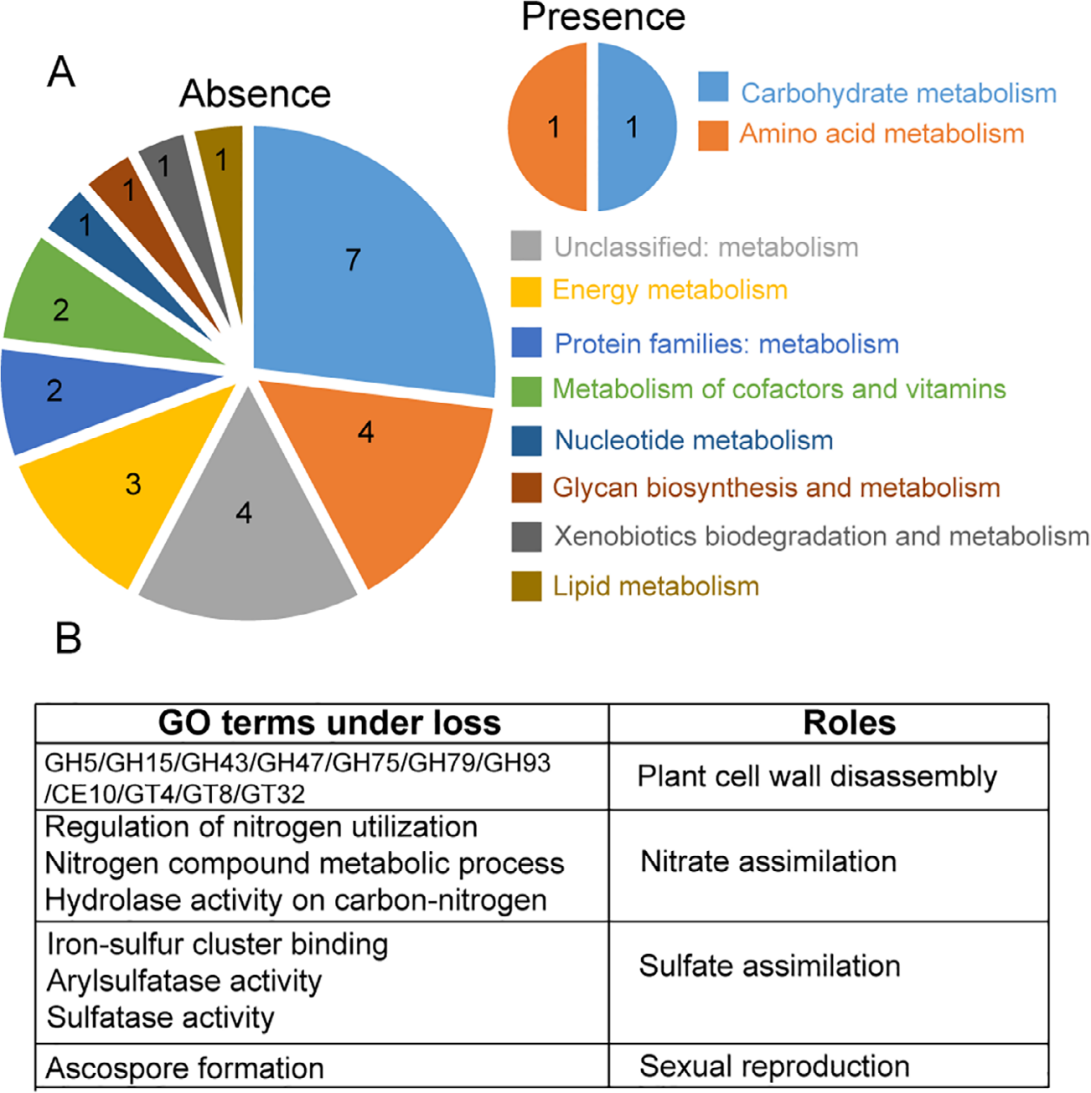
Genus‐specific present and absent genes in *Epichloë*. A. Metabolic pathways associated with genus‐specific absent versus genus‐specific present genes. The numbers in the piecharts represent the number of orthogroups. B. Four cases associated with genus‐specific absent genes. [Color figure can be viewed at wileyonlinelibrary.com]

Gene loss is primarily a feature of just one genus, *Epichloë* (Fig. 2B and C). To explore whether gene loss may be adaptive, we examined gene functions and found that *Epichloë* ‐specific absent genes are enriched for metabolic roles compared with present genes (Fig. 7A). Among *Epichloë*‐specific absent genes, CAZy genes and genes encoding enzymes in the nitrogen and sulfur assimilation are notably absent (Fig. 7B). These functions reflect an endophytic lifestyle where enzymatic degradation of the host is disadvantageous and resources, including nitrogen and sulfur compounds, can be co‐opted from the host. Together with massive gene loss (Fig. 2B), the genomic features of *Epichlöe* seem to be very close to biotrophic plant pathogens. In addition, we also find a genus‐specific absent gene that encodes a protein related to ascospore formation. It is well known that the production of ascospores represents the final step in sexual reproduction (Wilson *et al*., 2019), and although some of the species used here have both sexual and asexual forms (Tadych *et al*., 2014), we propose that the loss of this particular gene possibly coincides with the frequent occurrence of asexual *Epichloë* forms (Schardl *et al*., 2004).

### Genus‐specific selection also contributes to lifestyle transitions

**Fig 8:**
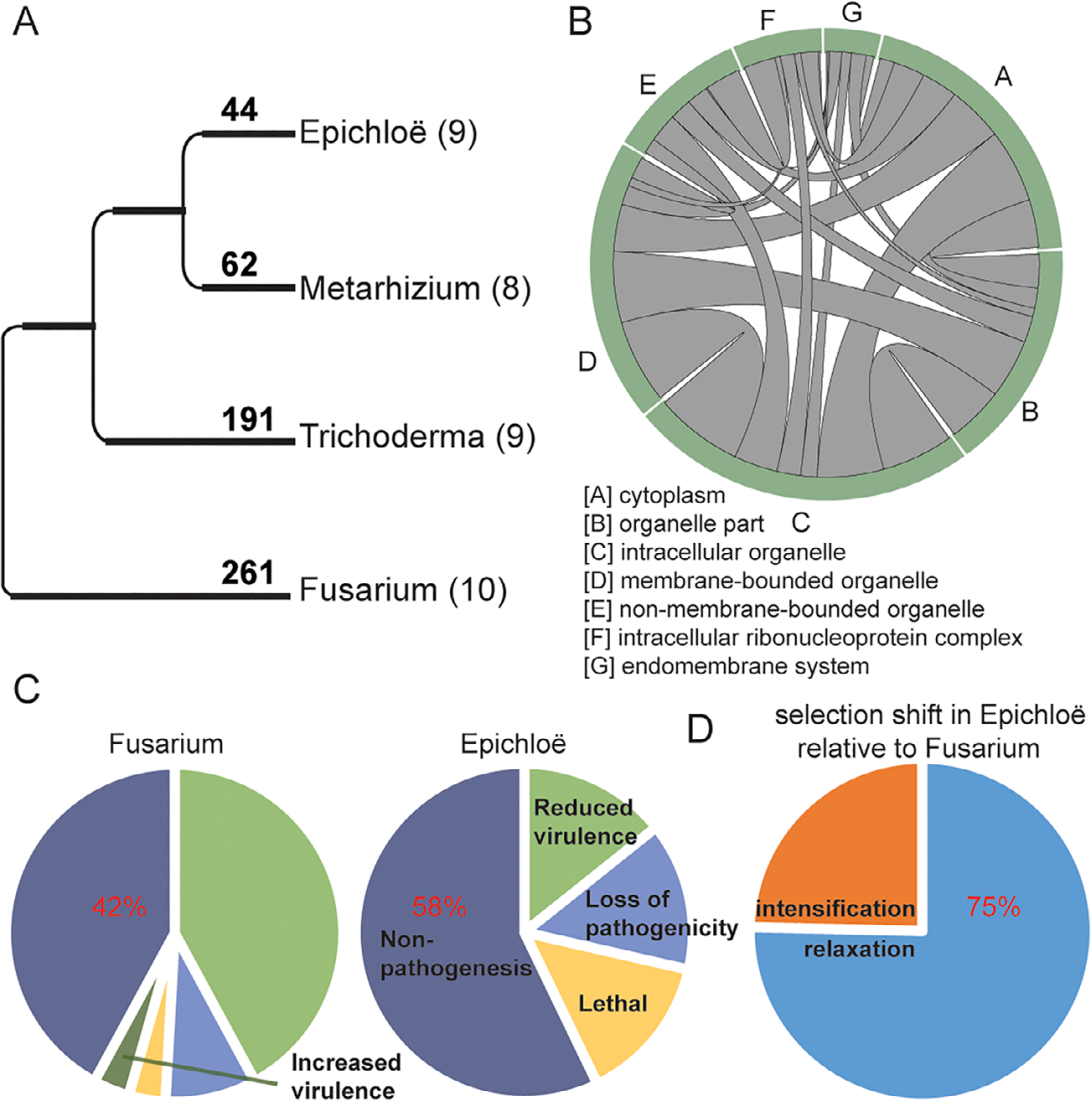
Positive and relaxed selection associated with lifestyle transitions. A. The numbers on the tree represent unique positive selection events at the branches leading to the four genera. Numbers in parentheses indicate the number of species studied in each genus. B. Subcellular location of positively selected genes in *Trichoderma*. C. Proportion of positively selected genes associated with pathogenesis. D. Selection shift in *Epichloë* relative to *Fusarium*. [Color figure can be viewed at wileyonlinelibrary.com]

Besides entire gene gain and loss, new functions can be established via amino acid changes driven by selection. To evaluate the role of selection in lifestyle emergence, we measure positive selection for each lifestyle using 2267 shared single‐copy genes across the four primary study genera. Sixty‐seven percent of positive selection events occurred once at the origin of specific lifestyles. Symbiotic *Epichloë* has the fewest positively selected genes, whereas pathogenic *Fusarium* has the most, but still only account for 11.5% of 2267 single‐copy genes (Fig. 8A and Table S7). In contrast to genus‐specific present genes, we find that positively selected genes more often function at the cytoplasm and membrane (Fig. 8B). The proportions among the four lifestyles suggest that positively selected genes are very likely correlated with intensity of pathogenesis. Through BLAST searches against the pathogen–host interactions (PHI) database (Urban *et al*., 2020), we further determined the roles of these selected genes in the two plant‐related genera. Among PHI hits, 57% (33 genes out of 57 hits) of positively selected genes in the plant pathogen *Fusarium* are related to pathogenesis, whereas plant symbiotic *Epichloë* has 42% (three genes out seven hits) of these genes related to pathogenesis (Fig. 8C). It is worth noting that genes in the PHI database have been classified on the basis of heightened virulence in other species, but likely have the inverse non‐virulence role in symbiotic *Epichloë*. Generally, these patterns may reflect a host‐fungi arms race in the pathogenic lifestyle versus little antagonism in the symbiotic lifestyle. We also estimate the selection strength in *Epichloë* relative to *Fusarium* using RELAX (Wertheim *et al*., 2015). In over half of the 2267 shared single‐copy genes (1602/2267 or 70%), we were able to detect differences (*P* < 0.05) in selective strengths. Again consistent with its endophytic lifestyle, *Epichloë* has more genes (75%) under relaxed selection than under intensified selection (Fig. 8D and Table S8).

## Discussion

### Contribution of gene turnover to new lifestyles

During evolution, organisms can gain new genes to perform new functions, recycle old ones to perform new functions and lose other genes that are not used anymore. Gene gains thus have been thought to play key roles in functional innovation. Many studies have evaluated the roles of gain and loss in evolution using the ratio of gain/loss at specific nodes (Paps and Holland, 2018; Shen *et al*., 2018; Bowles *et al*., 2020; Fernandez and Gabaldon, 2020). Recent studies comparing hundreds of plants and dozens of animal species have revealed genomic novelties (gene gains) associated with key evolutionary steps over 800 million years (Paps and Holland, 2018; Bowles *et al*., 2020). In this study, we study a key order in the third major kingdom – Fungi – to identify the important roles of genus‐specific genes in lifestyle transitions. We do so over a relatively short timescale, probably no more than 150 million years (Zhang *et al*., 2018b). It is worth emphasizing that not all genus‐specific gained genes in the *Hypocreales* order are strictly ‘new’ genes. Over 37% of genus‐specific present genes have homologues in other fungal orders. Three inter‐kingdom HGT cases were also found to contribute to the lifestyle of entomopathogenism and mycoparasitism/root symbiosis. For instance, the insect pathogen *Metarhizium* gained two new genus‐specific genes (MAN_05738 (trypsin‐like serine protease) and MAN_07610 (GH43)) from bacteria, which contribute to pathogenesis during insect infection (Fig. 3C).

On the other hand, some studies highlight that ‘loss is gain’, and that reductive evolution is a major contributor to evolutionary diversification, as is well documented for parasitic and symbiotic lineages (Vogel and Moran, 2013; Albalat and Canestro, 2016; Shen *et al*., 2018). This finding was expected. Symbiotic and parasitic lifestyles allow microbes to reside in plants and gain resources from its host, thus requiring fewer genes of their own (Duplessis *et al*., 2011; de Man *et al*., 2016; Xia *et al*., 2018). Coupled with this, previous studies indicate that losses of genes encoding CAZys and enzymes in the nitrogen and sulfur assimilation pathways enable biotrophs to adapt to plant hosts by avoiding recognition by the plant defence systems. In the *Hypocreales* order, we have shown that the endophytic lifestyle is associated with substantial gene loss in *Epichloë* (Fig. 7). Therefore, a newly established lifestyle is characterized by both gene gain and gene loss.

### Repeated gene gain and loss may be characteristic of recurrent lifestyle transitions

Lifestyles have evolved recurrently across the fungal tree of life, suggesting that lineages/genera frequently reconfigure their gene content to take advantage of open ecological niches. For instance, within and beyond the *Sordariomycetes* family, plant pathogenic, entomopathogenic and symbiotic lifestyles have been documented in different orders (Fig. 1; Shang *et al*., 2016; Zhang *et al*., 2018b). In this study, we show that fungi with similar lifestyles tend to share more lifestyle‐related orthogroups, although the shared proportion tends to be lower than 30% (Fig. 4), indicating that not all genus‐specific genes are required for a genus' lifestyle. In general, though, we propose that repeated gene gain and loss is characteristic of recurrent lifestyle transitions.

### Complementary roles of gene turnover and positive selection

Fungi can adapt to new lifestyles through the gain and loss of entire genes, such as genus‐specific present genes, or through amino acid changes, such as genus‐specific positively selected genes. We compare these roles and find a potentially complementary relationship. GO term enrichment analysis suggests that genus‐specific present genes tend to encode proteins that are located in extracellular zones (Fig. 5), whereas positively selected genes tend to encode proteins that function within the cytoplasm and membrane (Fig. 8). Correspondingly, genus‐specific genes more often degrade host walls (*Fusarium* and *Trichoderma*) or defence peptides made by the host (*Metarhizium*; Fig. 3), whereas positively selected genes are enriched in intracellular transport and metabolic roles (Table S7). These observations suggest that turnover and selection of lifestyle‐specific genes involve different gene sets and play complementary roles that cover both intercellular and extracellular activities.

### Unexpected positive selection patterns

Although we expect effector genes or extracellular proteins to be under positive selection given their direct interaction with hosts, GO enrichment analysis shows that positively selected genes are significantly enriched for cytoplasmic proteins and organelle membrane proteins (Fig. 8). One caveat is that the single‐copy genes used for selection analysis might be biased towards cytoplasmic and membrane proteins in our study because many of these are single‐copy, whereas effectors and related proteins often form large gene families. Nevertheless, similar patterns have also been reported in independent studies, such as in the plant pathogens *Sporisorium reilianum* and *Ustilago hordei* (Schweizer *et al*., 2018). Consistent with this view, intracellular changes of metabolism, such as mitochondrial β‐oxidation, influence virulence in the maize pathogen *Ustilago maydis* (Kretschmer *et al*., 2012), and therefore cytoplasmic and membrane proteins are reasonable targets for positive selection. However, it is unclear whether the positive‐selection patterns observed here are prevalent right across the fungi kingdom, and further analyses using more fungi with different lifestyles will be needed to shed light on this matter.

## Conclusion

Here, we address a long‐standing evolutionary question: how many genes are needed for adaptation to new lifestyles? We hypothesize that genetic features shared across all species with the same lifestyle, but absent in genera with alternative lifestyles, are more likely to be crucial to that lifestyle. Three lines of evidence support this hypothesis: (i) enriched GO terms of genus‐specific genes are associated with lifestyles; (ii) genus‐specific genes show up‐regulated expression in response to their respective hosts during interactions; and (iii) genus‐specific genes are often shared by fungi with similar lifestyles. We, therefore, propose that relatively few genes (1–8%) play key roles in lifestyle transitions. Further, most genus‐specific genes arose as new genes, while patterns of gene presence are largely driven by independent loss rather than horizontal gene transfer. Positive selection has more uneven importance among different lifestyles, contributing most to lifestyle transitions associated with intensity of pathogenesis. Genus‐specific genes are often enriched in the secretome, while positively selected genes are mainly located intracellularly. Together, the analyses presented here provide new insight into lifestyle transitions, and in particular, emphasize that relatively few core genes are needed for fungi to adapt to new environments.

## Experimental procedures

### Genomic data collection

Protein sequences and their corresponding coding sequences from 67 species were collected from publicly available fungal genomes belonging to the *Hypocreales* order and other fungi families (Table S1). The protein files were used to determine annotation completeness against BUSCO 3.1.0 (Waterhouse *et al*., 2018). Only genomes with <5% of missing genes against the *Sordariomycetes* database (3725 single copy genes) or *Pezizomycotina* database (3156 genes) were used. We selected 36 species across four genera in the *Hypocreales* order, with a minimum of eight species for each genus as our focal targets. We assumed that species in each genus share a common ancestor, and that retained genes are important for their common features. *Fusarium* species in this study cover all four main complexes in the genus (Aoki *et al*., 2014) and *Trichoderma* species cover all three main clades (Kubicek *et al*., 2019).

### Orthogroup identification and building the Hypocreales tree

Orthogroups were inferred using orthofinder 2.3.14 (Emms and Kelly, 2019) with BLASTP and an expectation value of 10^−3^. As a conservative approach, an inflation value of 1.5 was used in MCL 14–137 (Enright *et al*., 2002). Single‐copy genes across the 67 genomes were used for phylogeny construction with fasttree 2 (Price *et al*., 2010) and RAxML‐NG 1.01 (Kozlov *et al*., 2019). Alignments of each single‐copy gene were first generated, trimmed by trimAI with the automated1 option (Capella‐Gutierrez *et al*., 2009), and were then concatenated into a species alignment. Because topologies generated by fasttree 2 and RAxML‐NG are the same, only the ‘fast global’ bootstrap estimates by fasttree 2 were used.

### Identification of genus‐specific orthogroups

Genus‐specific present orthogroups were defined as orthogroups that are present in all species in a genus (or absent only once to allow for some level of error in genome assemblies) and absent in every other species from every other genus. Genus‐specific absent orthogroups were defined as orthogroups that are absent in all species in a genus and present in every other species (or absent only once) from every other genus.

### Origin of genus‐specific present orthogroups

To search for the possible origin of genus‐specific present orthogroups, we performed BLAST searches using genus‐specific gene sequences (E value 10^−3^) against 190 non‐*Hypocreales* genomes from *Sordariomycetes* downloaded from JGI (Grigoriev *et al*., 2014) (https://genome.jgi.doe.gov/portal/sordariomycetes/sordariomycetes.download.html) and against non‐redundant protein sequences through the NCBI BLAST webserver (https://blast.ncbi.nlm.nih.gov/Blast.cgi?PAGE=Proteins). The phylogenetic tree for the best HGT case was constructed using IQ‐TREE 1.6.11 (Nguyen *et al*., 2015) with the best substitution model and ultrafast bootstrap (*n* = 1000).

### Duplication analysis

Unique duplicates refer to duplicates present in all species (or absent only once) in a genus and absent in every other species from every other genus. We used the CAFÉ 5 software (Mendes *et al*., 2020) for computational analysis of gene family evolution, and then identified the expansion and contraction of gene families.

### Identification of positive selection at ancestral branches and relaxed selection in *Epichloë*

Only single‐copy genes across all species from the four tested genera were used, so that the same number of genes were examined for each genus. Codon alignment of each single‐copy gene was generated using pal2nal 14 (Suyama *et al*., 2006). The aBS‐REL method in HyPhy 2.5 with default parameters (Smith *et al*., 2015) was used to detect positive selection for each gene at branches leading to the four lifestyles (four branches tested). We only counted genes with corrected *P*‐values smaller than 0.05 as positively selected candidates. To reduce saturation at synonymous sites, positively selected genes with Ks > 3 were removed. We also compared selection strengths in *Epichloë* relative to *Fusarium* using the RELAX test (Wertheim *et al*., 2015). We only counted genes with *P*‐values smaller than 0.05 as candidates.

### Functional annotation

GO annotations and gene descriptions were obtained using PANNZER2 (Koskinen *et al*., 2015) and Wei2GO (Reijnders, 2020). KEGG orthology was analysed with GhostKOALA against the ‘genus_prokaryotes + family_eukaryotes’ database (Kanehisa *et al*., 2016). Agrigo 2 (Tian *et al*., 2017) was used to assess functional enrichment. Potential pathogenic proteins were identified using BLASTP (E value 10^−5^, 50% identity and max_target 1) against the PHI database v4.10 (6776 manually curated genes) (Urban *et al*., 2020). Carbohydrate‐active enzymes (CAZymes) were annotated using dbCAN2 (Zhang *et al*., 2018a) with the HMM search, DIAMOND and Hotpep.

### Differential expression analysis

Expression patterns in *Fusarium graminearum*, *T. virens* and *M. anisopliae* were extracted from previous studies (Brown *et al*., 2017; Malinich *et al*., 2019; Iwanicki *et al*., 2020), while expression pattern of *T. harzianum* and *E. festucae* were reanalysed from published raw data. Raw transcriptome reads were downloaded from the NCBI SRA database (see accession numbers in Table S1). The experimental conditions are described fully in those papers (Steindorff *et al*., 2014; Chujo *et al*., 2019). Reads were filtered and trimmed using seqtk trimfq (https://github.com/lh3/seqtk), and filtered reads from each library were aligned to the corresponding reference genome. Gene counts were generated using feature counts 1.6.3 (Liao *et al*., 2014) using the gff3 annotations and mapped bam files. Only uniquely mapped reads were counted. Differential expression between two conditions was determined with edgeR 3.24 (Robinson *et al*., 2010) using a false discovery rate (FDR) < 0.05 and fold change ≥ 2 as cutoff values.

## Supporting information

**Appendix S1.** Supporting Information.

**Table S1** Genome information of the 67 studied genomes and raw RNA‐seq data accession numbers of two species.

**Table S2** Gene families significantly contracted or expanded at branches leading to new lifestyles. Function was annotated using proteins from *Fusarium graminearum*.

**Table S3** Unique duplicates in each genus. Function was annotated using proteins from *Fusarium graminearum*

**Table S4** Genus‐specific present orthogroups for each genus.

**Table S5** Genus‐specific absent orthogroups for each genus.

**Table S6** GO enrichment analysis of genus‐specific present genes.

**Table S7** Positively selected genes at branches leading to new lifestyles.

**Table S8** Gene families under significantly relaxed and intensified selection.

## Data Availability

All genomic and RNA‐seq data used in this article are publicly available. Access details are listed in Table S1.
