# Supplementary material for: Comparative genomics reveals a core gene toolbox for lifestyle transitions in Hypocreales fungi
Source: Environ Microbiol. 2021 May 11;23(6):3251–64. doi: 10.1111/1462-2920.15554 (PMC8360070; doi:10.1111/1462-2920.15554)
Supplement: Supplementary file 1 — Appendix S1. Supporting Information. [file EMI-23-3251-s009.pdf]

Comparative genomics reveals a core gene toolbox for lifestyle transitions in Hypocreales fungi

Baojun Wu[1,2], Murray P. Cox[1,2*]

[1] Statistics and Bioinformatics Group, School of Fundamental Sciences, Massey University, Palmerston North 4410, New Zealand

[2] Bio-Protection Research Centre, Massey University, Palmerston North 4410, New Zealand

[*] Corresponding Author:

Murray Cox, Statistics and Bioinformatics Group, School of Fundamental Sciences, Massey University, Palmerston North 4410, New Zealand.

Email: m.p.cox@massey.ac.nz; Phone: +64 6 951 7747

ORCID ID: orcid.org/0000-0003-1936-0236

This file includes:

Table S1 to S8

Fig S1 to S2

Tables

Table S1. Genome information of the 67 studied genomes and raw RNA-seq data accession numbers of two species.

Table S2. Gene families under significantly contracted or expanded at branches leading to new lifestyles. Function was annotated using proteins from *Fusarium graminearum*.

Tables S3. Unique duplicates in each genus. Function was annotated using proteins from *Fusarium graminearum*

Table S4. Genus-specific present orthogroups for each genus.

Table S5. Genus-specific absent orthogroups for each genus.

Table S6. GO enrichment analysis of genus-specific present genes.

Table S7. Positively selected genes at branches leading to new lifestyles.

Table S8. Gene families under significantly relaxed and intensified selection.

Figures

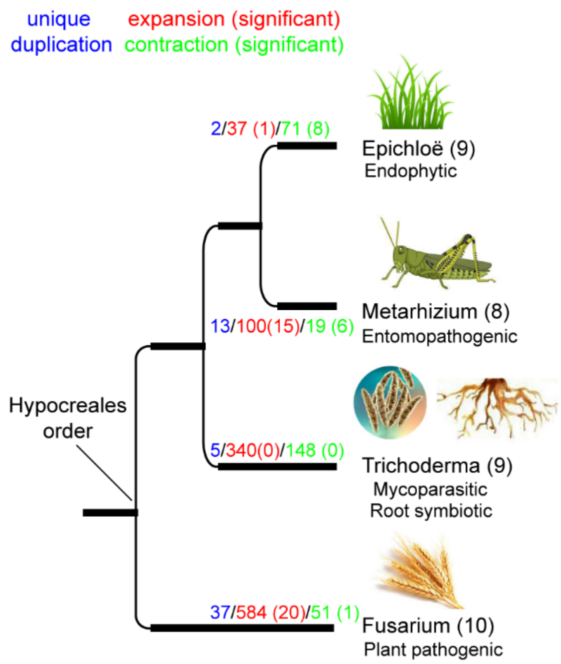

Figure S1. Gene family evolution in Hypocreales.

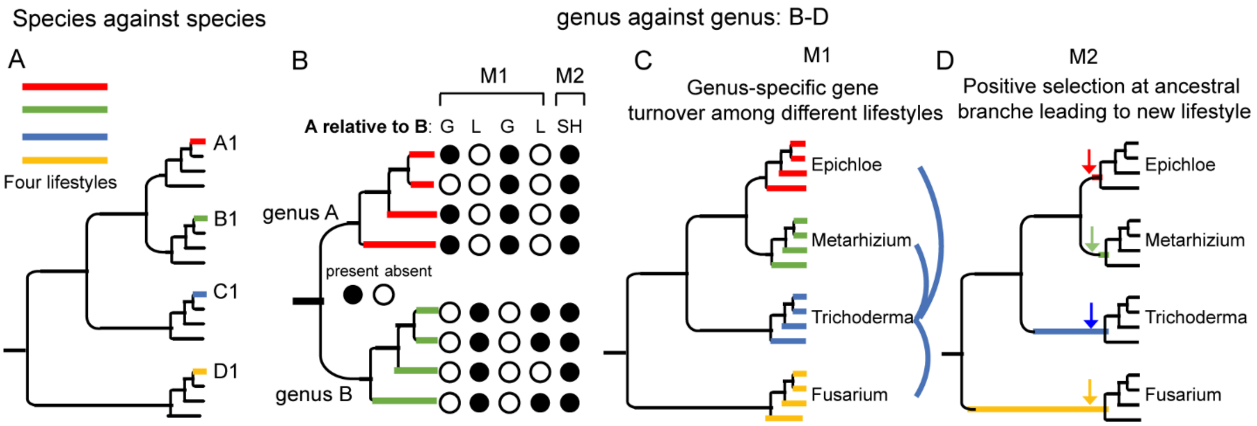

Fig. S2. Schematic representation of the study design. (A) A species method to identify genetic determinants of lifestyle. Four colors represent four different lifestyles. This method compares four individual species to identify lifestyle genes. (B) An example of employing a genus level method, which considers the entire gene set of all species of a genus, to study genus A-specific gain and loss relative to genus B. Columns 2,4 (L) and columns 1,3 (G) are loss cases and gain cases, respectively. The fifth column (SH) indicates genes present in all species in all genera that is used for genus-specific positive selection detection. (C) This study employs four genera to study genus-specific gene turnover. Figure C shows a case of '*Trichoderma*-specific gene turnover' only, where the genus *Trichoderma* is compared with the other three genera. (D) This study employs four genera to study genus-specific positive selection, where positive selection at each ancestral branch (location of arrow) is examined.
